# Epithelial rests of Malassez: from latent cells to active participation in orthodontic movement

**DOI:** 10.1590/2177-6709.22.3.119-125.sar

**Published:** 2017

**Authors:** Bianca Silva e Silva, Nathalia Carolina Fernandes Fagundes, Bárbara Catarina Lima Nogueira, José Valladares, David Normando, Rafael Rodrigues Lima

**Affiliations:** 1 Laboratory of Functional and Structural Biology, Institute of Biological Science, Universidade Federal do Pará (Belém/PA, Brasil).; 2 School of Dentistry, Universidade Federal de Goiás (Goiânia/GO, Brasil).; 3 School of Dentistry, Universidade Federal de Pará (Belém/PA, Brasil).

**Keywords:** Epithelial cells, Periodontium, Tooth movement.

## Abstract

**Introduction:**

The epithelial rests of Malassez (ERM) represent a group of cells in the periodontal ligament classically consisting of latent or quiescent structures associated with pathological processes. However, recent evidence shows that these structures cannot be considered only as cellular debris. The ERM is a major tissue structure, with functions in maintaining the homeostasis of periodontal tissue, including the maintenance of orthodontic movement.

**Objective:**

The present literature review aims at presenting the potential functions of ERM, with emphasis on orthodontic movement and the functional structure of the periodontium.

**Conclusion:**

ERM cells have a functional activity in modulation of orthodontic movement, trough their potential for differentiation, maintenance functions and the capacity of repairing periodontium.

## RESUMO

**Introdução:**

os remanescentes epiteliais de Malassez (REM) se configuram como um grupo de células epiteliais presentes no ligamento periodontal, classicamente consideradas estruturas latentes ou quiescentes, associadas a processos patológicos. Entretanto, ao longo dos anos, esse paradigma vem sendo rompido e hoje não mais são consideradas apenas como restos celulares, mas sim uma importante estrutura tecidual, com funções na manutenção da homeostase do periodonto, inclusive durante a movimentação ortodôntica.

**Objetivo:**

na presente revisão da literatura, buscou-se apresentar as funções dessas estruturas, com ênfase nelas durante a movimentação ortodôntica, rompendo o conceito errôneo de que são meros restos celulares, e defendendo sua compreensão como uma estrutura funcional do periodonto.

**Conclusão:**

os REM possuem uma atividade funcional na modulação da movimentação ortodôntica, por meio de seu potencial para diferenciação, de suas funções de manutenção e de sua capacidade de reparação periodontal.

## INTRODUCTION

For a long time, the scientific literature has not even hypothesized a role for the epithelial rests of Malassez (ERM). Initially, its latent or quiescent role was associated only to the formation of cysts and periapical granulomas. However, over the last 50 years, studies have gradually revealed the participation of ERM in the synthesis of mediators linked to the maintenance of periodontal homeostasis.^1^ ^,^ ^2^

The functional aspects of ERM are affected by mechanical forces in periodontal tissues during orthodontic tooth movement. Thus, this review aims at demystifying the idea that ERM constitute residual cells of odontogenesis with no defined function, but rather represent cells with responsibilities in maintaining periodontal space and orthodontic movement.

## FIRST STUDIES: MORPHOLOGICAL CHARACTERIZATION 

The ERM are resulting from cell fragmentation of the Hertwig epithelial root sheath (HERS), an apical extent of internal and external epithelial enamel, which stimulates the differentiation of ectomesenchymal cells into odontoblasts, secreting root dentin. Ectomesenchymal cells also induce the differentiation of the insertion periodontium. After these events, cells undergo fragmentation and remain as dispersed islands of epithelial cells in the periodontal ligament and eventually in the pulp.^3^ ^,^ ^4^

Initially described by Serres,^5^ these remaining cells of the periodontal ligament are portrayed as enamel organ debris. Decades later, Legros and Magiot^6^ pointed to the epithelial origin of these remaining cells and suggested that they could be associated with the formation of cysts, as dentigerous cysts and odontomas. Interestingly, Serres^5^ had approached the occurrence of HERS degeneration in the same work that described the enamel organ debris, but did not relate these events with each other.^5^ ^,^ ^6^

**Figure 1:**
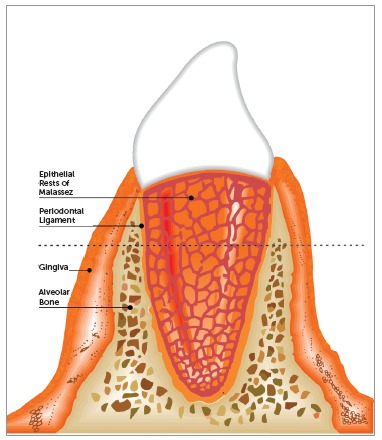
Morphology of ERM in human tooth: ERM distributed around the tooth root, forming something like a fishing net.

**Figure 2:**
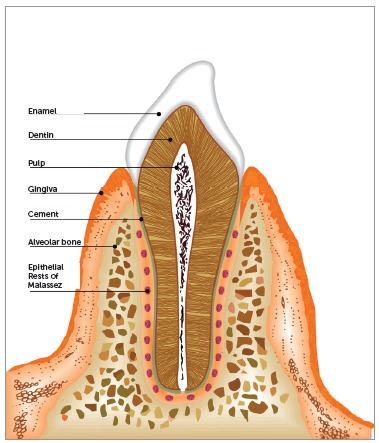
Conformation of ERM in a longitudinal slice in human tooth.

**Figure 3:**
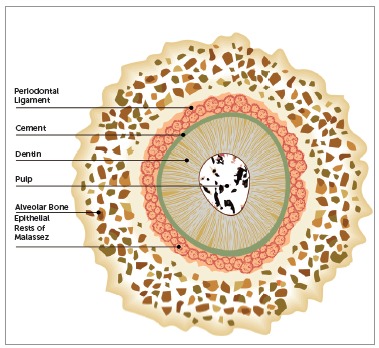
Scheme showing the conformation of ERM in longitudinal slice.

Malassez^7^ was the first to detail the histological characteristics of epithelial rests and their distribution in the periodontal ligament. In longitudinal and transverse slices of human specimens, Malassez noted the presence of epithelial cells that persisted around the roots (Figs 1, 2 and 3). After the first descriptions made by Malassez,^7^ other authors described the distribution of ERM and its relation to the dental element in a more detailed manner. These structures thus came to be known as the "epithelial rests of Malassez", receiving the eponymous name of its principal investigator.^7^

Subsequently, ERM were considered as lymphatic channels coated by epithelium and filled with lymph^8^. In subsequent research, Black^8^ started to understand them as glandular tissue,^8^ ^,^ ^9^ findings that have been proven wrong by recent histological studies.

Von Brunn, through the study of the development of the roots of teeth in rats, clarified the relationship between HERS and ERM.^1^ It was shown that the HERS consists of the union between the outer and inner epithelia of the enamel organ in the future region of the cementum-enamel junction.^1^ It was also suggested that ERM produce endocrine glands and hormones that would prevent the union of cementum to the alveolar bone.^9^ Orban^10^ stated that the epithelium rests contain a pseudotubular structure, indicating the possibility of endocrine function.

Regarding the location, the ERM often occurs in the periodontal ligament,^11^ ^,^ ^12^ with a predilection for the apex region, especially in the furca and cervical area of the tooth, in ascending order of frequency.^12^ Later studies also reported the presence of ERM in the pulp and cement.^13^ ^,^ ^14^ Wentz et al.^15^ analyzed the type and prevalence of ERM in the periodontal ligaments of rat molars. Three morphological variations were observed: small, proliferative and differentiated. Regarding prevalence, ERM were present in 50% of rats. ERM incidence decreased with increasing age. Regarding location, the results were similar to those reported by Reeve and Wentz:^16^ the supralveolar area (47%), middle third (30%), bifurcation region (15%) and apical region (8%). 

Reeve and Wentz^16^ determined the frequency of epithelial rests in patients aged 1-77 years, classifying different ERM types and locations on the periodontal ligament. The results showed that the ERM were present in all 31 specimens analyzed (regardless of age), but the incidence decreased with increasing age. As for the most frequent types of REM, small and differentiated ones decrease with age, while proliferative ones increase with age. Among young patients, most ERM were located in the cervical area and accumulated at the apex with increasing age. The authors suggested that ERM were persistent vestigial structures in the periodontal ligament with a potential role in the development of periodontal disease.^16^

Loe and Waerhaug^17^ were the first to suggest a compatible function for ERM, having described the spatial distribution of these structures around the tooth root. According to the authors, the ERM were not isolated structures, forming a structure similar to a fishing net around tooth roots (Fig 1), giving ERM a role in maintaining the periodontal space and preventing alveolodental ankylosis.^17^

Through scanning electron microscopy and immunohistochemistry, Ten Cate^18^ reported lower metabolic activity in cells of remaining epithelial components, suggesting a possible role of ERM should be disregarded in adult patients. However, later Trowbridge and Shibata^19^ identified mitotic activity in epithelium rests in experimental studies with animal models. Such evidence created possibilities for further investigations.

Using staining and electron microscopy to distinguish ERM in the surrounding region, Valderhaug and Zander^20^ noted the presence of numerous blood vessels between the REM and dental cementum, with a larger number of ERM close to the cementoenamel junction, without, however, touching the cementum. This result suggests the participation of ERM in the regeneration and repair of cementum.

Although some functions have been assigned to ERM by several authors, none has been supported by solid scientific evidence. The papers published to date only described its morphological characteristics. However, Ten Cate^21^ stated that ERM had a role in the formation of dental cysts, because the remaining epithelial cells from ERM behave the same way as other epithelial cells when the local tissue is changed. With support from deep periodontal ligament tissue, inflammation can proliferate within such tissue, giving rise to dental cysts.^21^

Brice et al^22^ published a study that contributed to change the paradigm of ERM as cell rests, reporting the presence of cells ultrastructurally similar to ERM in areas of root resorption with contiguous repair after orthodontic movement at the root surface of human premolars (in patients undergoing maxillary expansion). The ultrastructural characteristics of these cells provided evidence that they could be involved in mediating cementogenesis in the root resorption process, opening precedence for further research in this direction.^22^

## FUNCTIONAL CHARACTERISTICS OF EPITHELIAL RESTS OF MALASSEZ 

The ERM assists with the homeostasis of the periodontium, with a role in the renewal of collagen in the periodontal ligament, as well as the secretion of enamel proteins and bone matrix protein by these epithelial cells and specific enzymes.^23^ ^,^ ^24^ As part of this process, the ERM release inflammatory mediators such as prostaglandins and enamel proteins such as amelogenin and amelin. The latter also promote release of bone matrix proteins, BMP-2, osteopontin, osteoprotegerin and sialoprotein, proteins that aid in the repair and periodontal regeneration phase, aside from contributing to the repair of cementum itself.^25^ ^,^ ^26^ ^,^ ^27^

Enamel proteins have not limited participation in amelogenesis and are identified as active in inducing mantle dentin formation, the deposition of cellular and acellular cementum, cementum integrity maintenance, as well as a possible role as biological mediators.^13^ ^,^ ^28^ ^,^ ^29^ The presence of mechanical stresses along the periodontal ligament stimulates the release of bone matrix proteins, particularly osteopontin, which is associated with the prevention of ankylosis and root resorption.^22^ ^,^ ^27^ ^,^ ^30^ ^,^ ^31^ In this process, after the rupture of periodontal integrity, ERM release proteins of the enamel matrix and start to play a leading role in the early events of periodontal regeneration. The modulation of enamel protein expression by epithelial remnants in the ERM thus suggests inherent regenerative ability, through directed and controlled strategies.^32^ ^,^ ^33^

The bone morphogenetic proteins, especially BMP-2, as well as bone matrix proteins, act in regulating osteogenesis, with an initial rise in the dental follicle. These molecules have proven potential for bone remodelling throughout life.^34^ ^,^ ^35^

The prostaglandins, specifically prostaglandin E2, have a role in activating osteoclasts and can stimulate bone remodelling. Orthodontic treatment, in turn, is essentially a periodontal event that involves an acute inflammatory response during its initial phase. Furthermore, prostaglandins are important mediators of inflammation that play a key role in orthodontic movement, being synthesized by cyclooxygenases (COX). Prostaglandins stimulate bone resorption by increasing the number and activity of osteoclasts. Thus, prostaglandins have a major influence on the speed of orthodontic movement.^36^ ^,^ ^37^

In ERM, the release of epidermal growth factor (EGF) was verified as well.^1^ ^,^ ^38^ The EGF, compared to proximity to the periodontal apparatus, acts to lead resorption of the alveolar bone surface while keeping the human periodontal space in appropriate measure. The tooth movement induced by EGF causes an increase in periodontal tissue proliferation, which in turn is mediated by the continuous release induced by ERM. EGF plays an important role in the prevention of ankylosis and osteoclastogenesis and cementogenesis processes.^1^ ^,^ ^36^ ^,^ ^38^ Thus, during orthodontic treatment, there is no ankylosis, for the orthodontic movement does not promote the destruction of ERM. EGF also stimulates ERM to proliferate and maintain their organizational structure.^39^

Regarding the role of these receptors in osteoclastogenesis, Yi et al^40^ demonstrated the inhibition of the signalling pathways affected in this process, which is also mediated by the receptor activator of nuclear factor kappa-B ligand (RANKL) and causes apoptosis through caspase activation.^40^

From their potential in the context of maintaining periodontal insertion through the release of inflammatory mediator structures, proteins and growth factors, the ERM were identified as key figures in maintaining homeostasis, assisting in the maintenance of space in the periodontal ligament, cementum repair and regeneration processes and collagen renewal.^1^ ^,^ ^14^

### Maintenance of the periodontal ligament space 

The ERM represent the only odontogenic epithelial structure able to persist in the periodontal ligament in adulthood, as that is the only structure able to persist in situations in which the periodontal ligament tissue is affected. These resistant structures are extremely important for maintaining homeostasis. ERM are able to promote the maintenance and regeneration of the periodontal ligament space.^41^ ^,^ ^42^

This process is triggered by EGF and prostaglandins released from bone resorption of the alveolar surface and preserves the human periodontal space, which varies in thickness from 0.20 to 0.40 mm.^1^ Such mediators have been shown to be important in the bone resorption process, thus contributing to maintenance of the periodontal ligament. 

The regeneration of periodontal tissue occurs through assistance in the renewal of cells that suffered some sort of damage. Damage to periodontal tissue is directly associated with orthodontic movement, where hyalinizing outbreaks have been recorded on the pressure side in the periodontal ligament, mainly during the first hours after the application of force.^43^ ^,^ ^44^

ERM are structures that maintain the balance of the environment, contributing to the regeneration of the cementoblastic process. Unlike bone cells, cementoblasts do not have receptors for remodelling mediators, requiring the beginning of a cascade of events that causes the pH to normalize. This promotes the inclusion of new collagen fibres at the damaged cementoblastic surface, induced by ERM, allowing the root surface to regenerate and return to its normal state.^27^ ^,^ ^36^

The maintenance of this balance by REM allows for maintenance of the periodontal ligament space^45^ ^,^ ^46^ and prevents the occurrence of dentoalveolar ankylosis, contributing to reorganization of the periodontal ligament during orthodontic tooth movement and resorption.^47^

### Regeneration and repair of cement

From the release of substances and differentiation of its epithelial cells, the remaining ERM play an important role in the formation and repair of cementum, particularly in the differentiation of cementoblasts with mesenchymal origin, preferably through the direct transformation of epithelial cells to cementoblasts.^1^ ^,^ ^48^ ^,^ ^49^

This role is contrary to the events resulting from the induced orthodontic tooth movement. After the application of force, the change in the levels of periodontal capillary pressure can trigger blood supply dysfunction until its collapse, according to the force applied. This process can result in cementoblast degradation and cementoid tissue layer formation.^50^

According to Hasegawa et al,^25^ the ERM are related to the repair of cementum due to the release of enamel matrix proteins expressed during tooth development, which is directly associated with epithelial-mesenchymal interactions. In this procedure, epithelial cells associated with ERM modify their distribution and express BMP-2, osteopontin and ameloblastin. 

Cementoblasts are involved in the process of remodelling and repair of the root surface.^51^ The application of mechanical forces to the tooth occurs based on changes in vascular flow and the synthesis of prostaglandins, cytokines and growth factors. The actions of these mediators result in bone remodelling, which involves resorption or bone deposition on opposite side of the periodontal ligament.^52^ ^,^ ^53^

### Prevention of alveolodental ankylosis

Alveolodental ankylosis is characterized by the absence of tooth elastin due to the loss of periodontal ligament cells associated with root resorption.^27^ This situation occurs after the removal of ERM, followed by the resorption of dental tissue, which is resorbed and replaced by bone. ERM keep the bone away from the root surface through the release of mediators such as EGF, which induces osteoclasia. In cases of trauma, where the ERM are damaged, leading to necrosis, mediators that promote osteoclasia may not be present in the required concentration, which, as a result, may lead to alveolodental ankylosis.^54^ ^,^ ^55^

Orthodontic movement, however, does not promote ERM necrosis, but, rather, just the reverse. When properly executed, the orthodontic movement is able to stimulate the secretory power of these cells, which prevents the recurrence of ankylosis. The growth in size of ERM during tooth movement indicates the level of the response to mechanical stimulation and its possible role in tooth remodelling.^36^ ^,^ ^37^ ^,^ ^47^ ^,^ ^56^

### Collagen renewal

Collagen plays a key role in supporting the tensile forces; periodontal ligament compression and its renewal are extremely important in maintaining the ligament space and protecting the tissue from mechanical forces. ERM have been associated with the induction of this process from latent collagenase secretion in the extracellular matrix, allowing for the transformation of active collagenase enzymatic cleavage, resulting in the synthesis of collagen fibres.^43^ ^,^ ^52^ ^,^ ^53^

The synthesis of collagen as well as changes in the matrix of the periodontal ligament contribute to the bone remodelling process, which induces orthodontic forces^57^ ^,^ ^58^. During the early inflammatory events that compose the induced movement process, the constriction of the microvasculature of the periodontal ligament results in focal areas of necrosis, with histological features of hyalinization.^34^ ^,^ ^59^ During this process, the synthesis of new collagen, in a process induced by ERM, promotes homeostasis refurbishment and the maintenance of periodontal tissue.^52^

## CONCLUSIONS

The ERM cells indicated to present many functions at tooth movement after orthodontic forces. The capacity to repair periodontium and to prevent cementum damages were connected with this process. Moreover, an increase in ankylosis events related to the processes and the hyalinization of periodontal tissue after the application of mechanical forces during the aging process can be associated to the potential of ERM. Otherwise, more studies are necessary to clarify these functions and consequences in daily orthodontics practice.
